# Molecular principles of recruitment and dynamics of guest proteins in liquid droplets

**DOI:** 10.1038/s41598-021-98955-0

**Published:** 2021-09-29

**Authors:** Kiyoto Kamagata, Nanako Iwaki, Milan Kumar Hazra, Saori Kanbayashi, Trishit Banerjee, Rika Chiba, Seiji Sakomoto, Virginie Gaudon, Bertrand Castaing, Hiroto Takahashi, Michiko Kimura, Hiroyuki Oikawa, Satoshi Takahashi, Yaakov Levy

**Affiliations:** 1 Institute of Multidisciplinary Research for Advanced Materials, Tohoku University, Katahira 2-1-1, Aoba-ku, Sendai, 980-8577 Japan; 2 Department of Chemistry, Graduate School of Science, Tohoku University, Sendai, 980-8578 Japan; 3 Graduate School of Life Sciences, Tohoku University, Katahira 2-1-1, Aoba-ku, Sendai, 980-8577 Japan; 4 Department of Structural Biology, Weizmann Institute of Science, 76100 Rehovot, Israel; 5 Department of Synthetic Chemistry and Biological Chemistry, Graduate School of Engineering, Kyoto University, Katsura, Nishikyo-ku, Kyoto, 615-8510 Japan; 6 Centre de Biophysique Moléculaire, CNRS, UPR4301, rue Charles Sadron, 45072 Orléans, France

**Keywords:** Computational biophysics, Intrinsically disordered proteins, Single-molecule biophysics

## Abstract

Despite the continuous discovery of host and guest proteins in membraneless organelles, complex host–guest interactions hinder the understanding of the molecular grammar governing liquid–liquid phase separation. In this study, we characterized the localization and dynamic properties of guest proteins in liquid droplets using single-molecule fluorescence microscopy. Eighteen guest proteins of different sizes, structures, and oligomeric states were examined in host p53 liquid droplets. Recruitment did not significantly depend on the structural properties of the guest proteins, but was moderately correlated with their length, total charge, and number of R and Y residues. In contrast, the diffusion of disordered guest proteins was comparable to that of host p53, whereas that of folded proteins varied widely. Molecular dynamics simulations suggest that folded proteins diffuse within the voids of the liquid droplet while interacting weakly with neighboring host proteins, whereas disordered proteins adapt their structures to form tight interactions with the host proteins. Our study provides insights into the key molecular principles of the localization and dynamics of guest proteins in liquid droplets.

## Introduction

Recent evidence suggests that membraneless organelles, such as stress granules and nucleoli, are liquid droplets formed by liquid–liquid phase separation (LLPS)^1–5^. LLPS-relevant molecules, including proteins, RNA, and DNA, concentrate within the liquid droplets and move inside these droplets, which work as reaction fields for various biological functions. LLPS-relevant proteins such as LAF-1, FUS, and TDP-43 have recently been identified. We have previously demonstrated that the tumor suppressor protein p53 itself forms liquid droplets^6^. LLPS-relevant proteins are categorized into host (or scaffold) and guest (or client) proteins. Host proteins can form liquid droplets while guest proteins are localized in the liquid droplets.

Despite the continuous discovery of LLPS-relevant proteins, complex host–guest interactions hinder the understanding of the molecular grammar governing LLPS. For natural host proteins, intrinsically disordered proteins (IDPs), rather than folded proteins, tend to form liquid droplets via multivalent intermolecular interactions, such as cation–π, hydrophobic (including π–π), and electrostatic interactions^6–12^. In contrast, guest proteins differ between membraneless organelles^3^ and the localization signal, identified in membrane-bound organelles, has not been clearly identified for guest proteins, except for specific host–guest interactions^13^. Nott et al. examined the recruitment of several proteins in Ddx4 droplets and found that the relative contents of R, Y, and P residues in guest proteins, rather than isoelectric point (pI) and molecular weight, correlated with the recruitment tendency^14^. Similarly, Wang et al. investigated the localization of various FUS family proteins in FUS droplets and found that the number of R and Y residues in the guest proteins correlated with the recruitment tendency^10^. These data indicate the importance of cation–π interactions in the recruitment, but with a difference between the absolute and relative residue numbers. π–π and electrostatic interactions, identified between host protein molecules, may participate in the recruitment capability of guest proteins. Comparisons between host and guest proteins have demonstrated that the amino acid components of guest proteins are more similar to those of the folded protein than those of IDPs^7^, implying a structure-dependent recruitment property for the guest proteins.

Another molecular property is the dynamics of the liquid droplets. Protein diffusion in dilute solutions depends on the molecular size and viscosity following the Stokes–Einstein equation. In contrast, guest protein dynamics in liquid droplets are more complicated than those in dilute solutions. In droplets, the excluded volume effect slows the dynamics of guest proteins, as shown in 4E binding protein 2 within Ddx4 droplets^8^. In addition, the pattern of interaction between a guest protein and its neighboring host proteins varies with time. Furthermore, the host proteins are distributed non-uniformly at the microscopic level, and voids exist inside the droplets^15–17^. The dynamics in the droplets have been characterized using fluorescence recovery after photo-bleaching^18–21^, fluorescence correlation spectroscopy^15,22^, and single-molecule fluorescence microscopy^23^. Two proteins exhibited different biphasic dynamics in stress granules^23^. Hopping diffusion has also been proposed^24^. The complicated dynamic behavior of guest proteins is expected due to several intermolecular interactions with non-uniformly distributed host proteins.

In this study, we aimed to characterize the recruitment and dynamic properties of guest proteins of different sizes, structures, and oligomeric states in liquid droplets. We examined 18 fluorescent dye-labeled or green fluorescent protein (GFP)-fused guest proteins (including mutants) in host p53 liquid droplets using single-molecule fluorescence microscopy. We found similar recruitment properties but different dynamic properties of the folded and disordered guest proteins. Furthermore, we used molecular dynamics (MD) simulations to understand the heterogeneity in the dynamic behavior of guest proteins.

## Results

### At least four droplet-forming disordered p53 domains are required for high uptake into p53 tetramer droplets

We examined guest protein uptake into host protein liquid droplets. We used the tumor suppressor protein p53 as the host because multivalent interactions between disordered p53 domains are a common property in droplet-forming proteins^6^. The thermostable and single cysteine mutant of p53 was used because it has less solid-aggregation capability than the wild type^25^. We used several proteins with different sizes, structures, and oligomeric states as guests. These guest proteins, except p53 and its mutants, did not strongly interact with the host p53 under the no-droplet condition, as no significant increase in fluorescence anisotropy of labeled guest proteins was observed upon addition of the non-labeled p53 (Supplementary Fig. S1).

**Figure 1:**
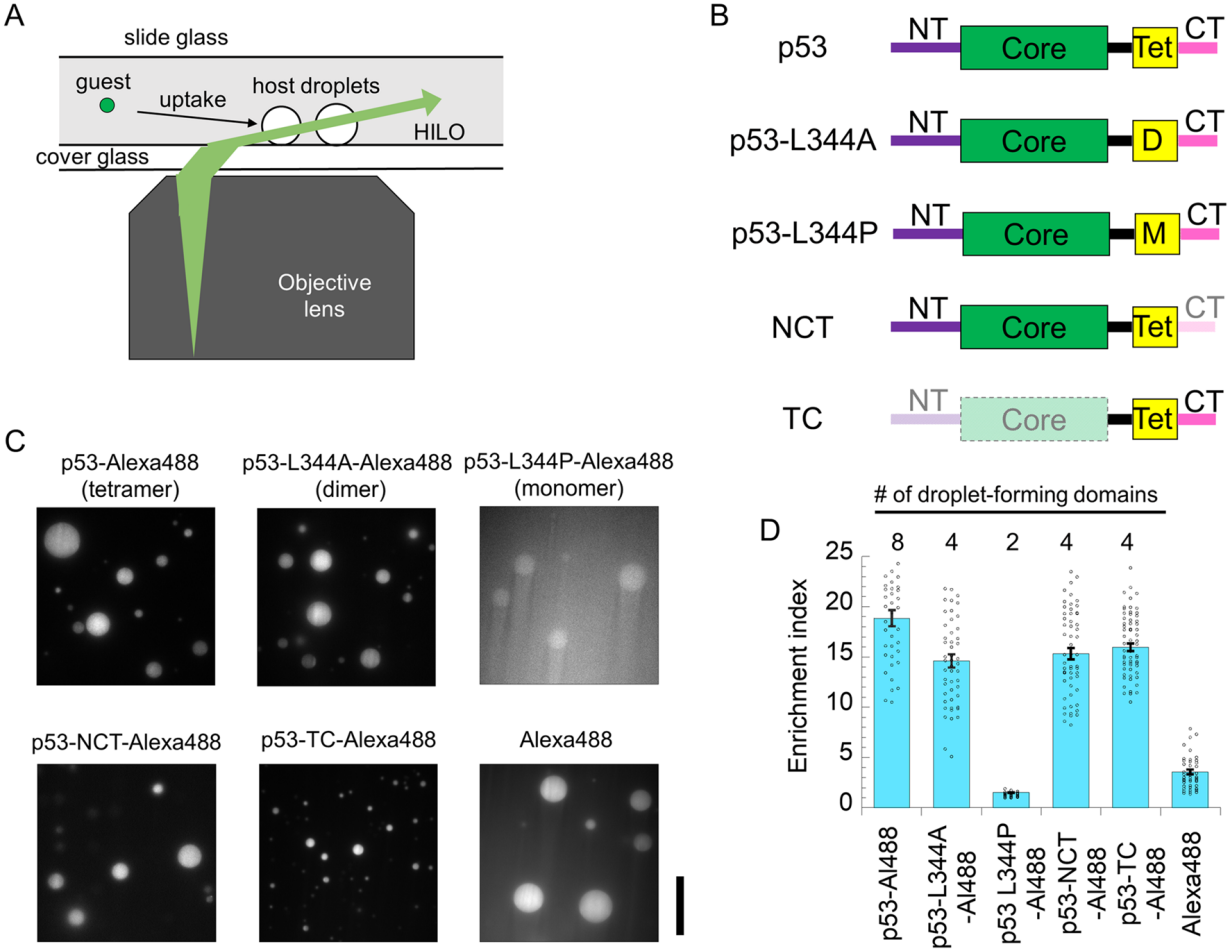
At least four sets of droplet-forming disordered p53 domains are required for recruitment into liquid p53 tetramer droplets. (**A**) Schematic diagram of fluorescent microscopic measurements with highly inclined and laminated optical sheet (HILO) illumination for labeled guest proteins in non-labeled host protein droplets. (**B**) p53 mutants used in the study. NT, core, Tet, and CT represent the N-terminal, core, tetramerization, and C-terminal domains of p53, respectively. D and M represent dimer and monomer mutations in Tet domain, respectively. Green and yellow boxes represent folded regions, whereas the other colors (purple, black, and pink) represent disordered regions. (**C**) Fluorescent images of Alexa488-labeled p53 mutants with different oligomeric states in non-labeled p53 tetramer droplet solution. Scale bar denotes 20 μm. (**D**) Enrichment index (EI) of the guest p53 mutants into non-labeled p53 tetramer droplet. Dots represent the average EIs of each droplet. The errors denote the standard errors. Al488 denotes Alexa488. # of droplet-forming domains denotes the number of NT plus CT. Significant differences in average EI values between p53-Alexa488 and four p53 mutants were confirmed using Welch’s t test with α = 0.05. Significant differences were also confirmed in average EI values between Alexa488 and five p53 samples.

We first demonstrated Alexa488-labeled p53 tetramer uptake into the non-labeled p53 tetramer droplets using fluorescence microscopy with highly inclined and laminated optical sheet (HILO) illumination (Fig. 1A). The primary structures of p53 and its mutants (used in subsequent experiments) are described in Fig. 1B. The concentrations were set to 0.1 μM for the labeled guests and 25 μM for the non-labeled host. The fluorescence intensity of the droplets was, on average, 18.9-fold higher than that of the solution (Fig. 1C). The enrichment index (EI), calculated from the fluorescence intensity in the droplets divided by that in the solution, was used as the uptake indicator (Fig. 1D). Next, we examined the effect of the oligomeric state on uptake into the p53 tetramer droplets (Fig. 1B–D). We prepared Alexa488-labeled p53 dimer (L344A)^26^ and monomer (L344P)^27–29^ mutants. The average EI of the p53 dimer mutant decreased slightly to 14.6. In contrast, the average EI of the p53 monomer mutant decreased significantly to 1.5, which was comparable to that of Alexa488 (average EI: 3.6). Considering that the p53 tetramer forms droplets via interactions of four sets of N- and C-terminal domains^6^, the reduced number of sets (two domains) weakens the recruitment capability.

Next, we investigated the effect of removing either the droplet-forming disordered domains (N- and C-terminal domains) of p53 on uptake into the p53 tetramer droplets. These two domains have different molecular properties: negative charge-rich 95 residues in the N-terminal domain and positive charge-rich 36 residues in the C-terminal domain. We prepared NCT and TC mutants of p53, which lack the C-terminal domain and N-terminal plus core domains, respectively, maintaining a tetramer (Fig. 1B)^30–32^. The average EIs of the NCT and TC mutants were slightly lower than that of the p53 tetramer but were comparable to that of the dimer mutant (Fig. 1C,D). This suggests the importance of the number of droplet-forming domains of guest p53, regardless of the domain composition, on recruitment. Taken together, at least four droplet-forming domains are required for high recruitment.

### The recruitment tendency of guest proteins does not depend on their structural properties

**Figure 2:**
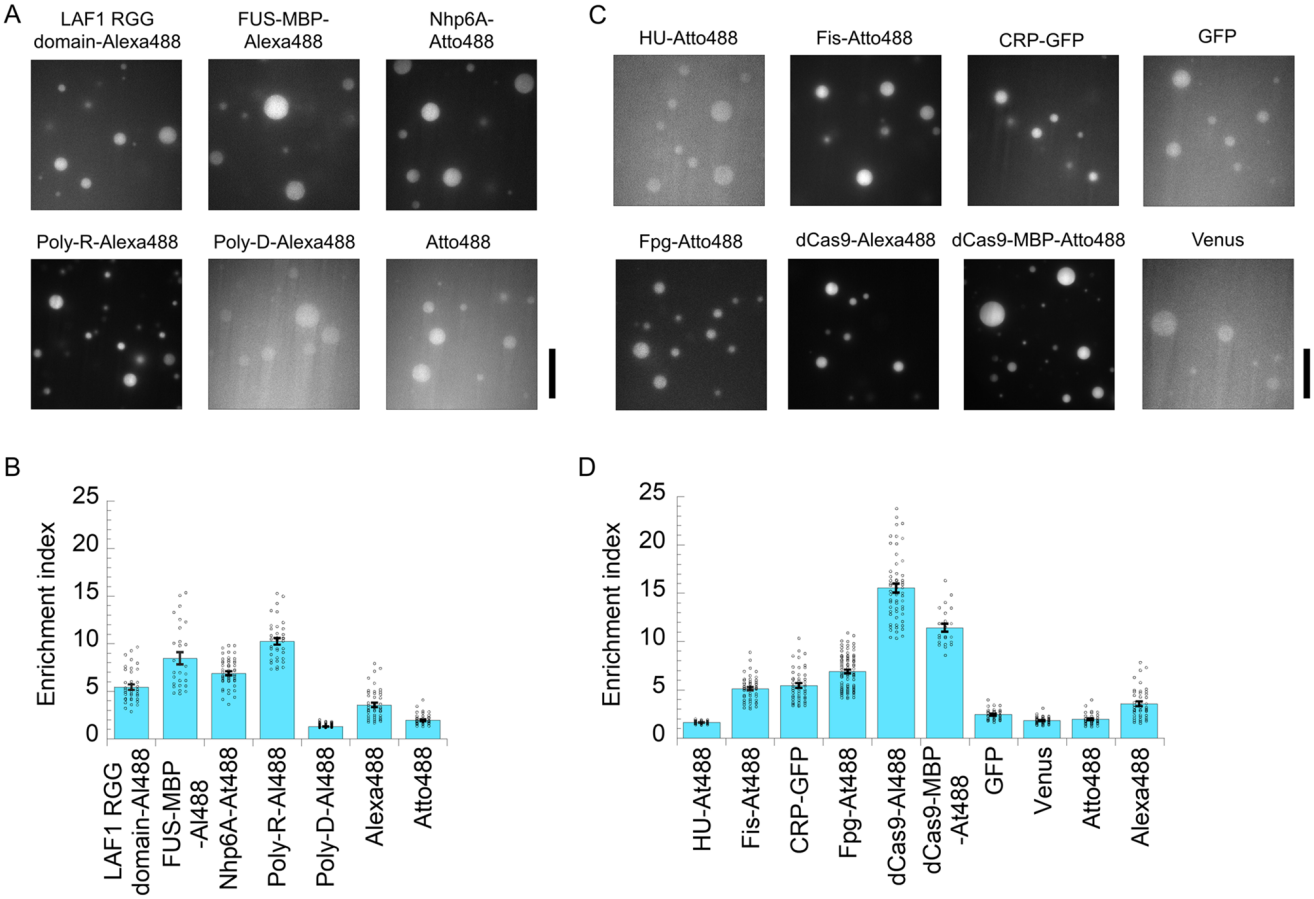
Structural properties of guest proteins do not determine recruitment into liquid p53 tetramer droplets. (**A**) Fluorescent images of labeled intrinsically disordered proteins (IDPs) and artificial polymers in non-labeled p53 tetramer droplet solution. Alexa488-labeled LAF-1 RGG domain, FUS–Alexa488, Nhp6A–Atto488, Poly-R–Alexa488, and Poly-D–Alexa488 are displayed. (**B**) Enrichment index (EI) of the guest IDPs and artificial polymers into non-labeled p53 tetramer droplet. At488 and Al488 denote Atto488 and Alexa488, respectively. Significant difference of the average EI values for all IDPs and artificial polymers from Alexa488 or Atto488 was confirmed using Welch’s t test with α = 0.05. (**C**) Fluorescent images of labeled folded and fluorescent proteins in non-labeled p53 tetramer droplet solution. HU–Atto488, Fis–Atto488, CRP–GFP, FPG–Atto488, dCas9–Alexa488, dCas9–MBP–Atto488, GFP, and Venus are displayed. (**D**) EI of the guest folded proteins into non-labeled p53 tetramer droplet. Scale bars in panels B and D denote 20 μm. The errors in panels B and D denote the standard errors. Significant difference of the average EI values for all folded proteins, except for CRP, from Alexa488 or Atto488 was confirmed using Welch’s t test with α = 0.05. Similarly, significant difference of the average EI values between CRP-GFP and GFP was confirmed.

Since many IDPs participate in droplet formation, we hypothesized that they may be recruited into the droplets more efficiently than folded proteins. To test this hypothesis, we first examined the uptake of several charge-rich IDPs into the p53 tetramer droplets (Fig. 2A,B). The RGG domain of LAF-1 (fully disordered) and maltose binding protein (MBP)-conjugated RNA-binding protein FUS (low-complexity disordered domain and partially structured RNA-binding domain) are known to form droplets by themselves^15,33^. These Alexa488-labeled proteins show moderate recruitment (average EI = 5.4 for RGG domain of LAF-1 and 8.5 for FUS–MBP). Moderate uptake was also observed in the Atto488-labeled DNA-binding protein Nhp6A (N-terminal disordered region and globular HMGB domain; average EI = 6.9). As a control, Atto488 itself exhibited lower recruitment (average EI = 2.0) than the labeled proteins. The positively charged Alexa488-labeled poly-R peptide (with a median of 200 residues) showed moderate recruitment (average EI = 10.2). In contrast, the recruitment of negatively charged Alexa488-labeled poly-D peptide (with a median of 200 residues; average EI = 1.3) was slightly lower than that of Alexa488. The comparison between poly-R and poly-D suggested the importance of the amino acid components of guest IDPs in recruitment. These results demonstrate the low and moderate uptake capabilities of IDPs.

Next, we investigated the uptake of several charge-rich folded proteins into the p53 tetramer droplets (Fig. 2C,D). Atto488-labeled dimeric DNA-binding proteins, HU and Fis, showed low and moderate recruitment, respectively (average EI = 1.6 and 5.1 for HU and Fis, respectively). The dimeric DNA-binding cAMP receptor protein (CRP) conjugated to GFP exhibited moderate recruitment (average EI = 5.5). In addition, the Atto488-labeled monomeric DNA-binding protein, Fpg, was moderately recruited (average EI = 6.9), while the Alexa488-labeled monomeric DNA-binding protein, dCas9 (deactivated mutant lacking the ability to cleave DNA), showed high recruitment (average EI = 15.5). The recruitment of Atto488-labeled MBP-conjugated dCas9 (dCas9-MBP) decreased slightly compared with that of dCas9 without MBP (average EI = 11.4). Furthermore, the fluorescent proteins GFP and Venus exhibited low uptake (average EI = 2.4 and 1.8 for GFP and Venus, respectively). Taken together, these results show that folded proteins have a wide range of recruitment capabilities. These results do not support the hypothesis that IDPs have a higher recruitment capability into droplets than folded proteins.

### Molecular interactions between the host and guests determine recruitment

**Figure 3:**
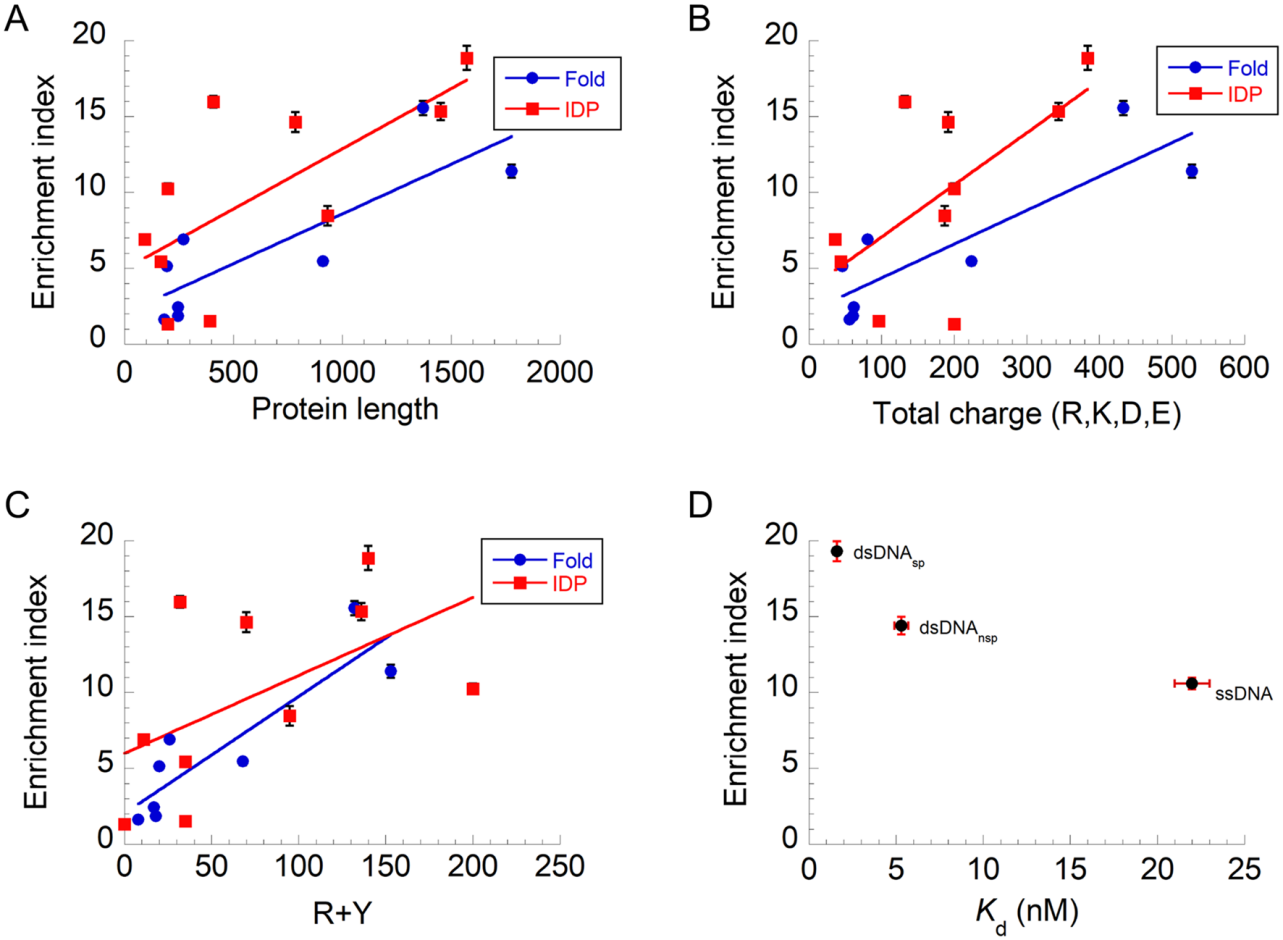
Molecular interaction between host and guests affects recruitment into liquid p53 tetramer droplets. (**A**) Protein length dependence of enrichment index (EI) of guest proteins. (**B**) Total charge dependence of EI of guest proteins. (**C**) EI of guest proteins against a function of R plus Y number. (**D**) EI of DNAs against a function of dissociation constant of DNAs to p53 tetramer. In all panels, errors denote standard errors. In panels A–C, best-fitted lines for the guest folded proteins (red) and intrinsically disordered proteins (IDPs) (blue) are displayed as a guide for eye.

We elucidated the molecular interactions between host and guest proteins by comparing the EI values and three physical parameters of the guest proteins, namely, protein length, total charge number (R, K, D, and E), and R plus Y residue number, corresponding to the guest protein size, the electrostatic interactions between the host and guests, and the cation–π interactions between the host and guests, respectively (Fig. 3A–C and Table 1). The EI values increased gradually for all datasets as the parameters increased, and no significant differences were detected between the folded and disordered proteins due to large deviations in the individual data. The correlation coefficients between EI and protein length, total charge number, and R plus Y residue numbers were 0.69, 0.66, and 0.68, respectively, indicating moderate correlations. In contrast, the relative contents of charges and R plus Y residues showed no significant correlations with the EI values (*r* = 0.12 and –0.14), suggesting the impact of absolute number of uptake-relevant residues. In contrast, the net charge of guest proteins was not correlated with the EI values (*r* = 0.22). Therefore, electrostatic and cation–π interactions participate in guest protein recruitment.

**Table 1:** Enrichment indices, physical parameters, and diffusion coefficients of guest proteins in the droplets of p53 tetramer.

Guest proteins	Enrichment index	*D* (μm^2^ s^−1^)	Length (AA)	Total charge *	R + Y
**IDPs**					
p53 tetramer	18.9 ± 0.8	0.031 ± 0.002	1572	384	140
p53 L344A (dimer)	14.6 ± 0.7	0.039 ± 0.002	786	192	70
p53 L344P (monomer)	1.51 ± 0.04	0.046 ± 0.004	393	96	35
p53 NCT (tetramer)	15.3 ± 0.6	0.026 ± 0.003	1452	344	136
p53 TC (tetramer)	16.0 ± 0.4	0.054 ± 0.006	408	132	32
RGG domain of LAF-1	5.4 ± 0.3	0.071 ± 0.003	168	44	35
FUS-MBP	8.5 ± 0.6	0.051 ± 0.005	934	187	95
Nhp6A	6.9 ± 0.2	0.048 ± 0.004	93	36	11
poly-Arg	10.2 ± 0.3	0.036 ± 0.003	200	200	200
poly-Asp	1.31 ± 0.02	0.052 ± 0.003	200	200	0
**Folded proteins**					
HU (dimer)	1.63 ± 0.02	0.43 ± 0.06	184	56	8
Fis (dimer)	5.1 ± 0.2	0.93 ± 0.08	196	46	20
CRP-GFP (dimer)	5.5 ± 0.2	0.19 ± 0.02	912	224	68
Fpg	6.9 ± 0.2	1.31 ± 0.09	272	81	26
dCas9	15.6 ± 0.5	0.070 ± 0.004	1371	433	132
dCas9-MBP	11.4 ± 0.4	0.048 ± 0.003	1779	528	153
GFP	2.4 ± 0.1	1.67 ± 0.08	246	62	17
Venus	1.84 ± 0.06		246	61	18

*The total charge number was calculated as the number of R, K, D, and E.

To confirm the relationship between recruitment capability and host–guest interaction, we measured the uptake of three DNA fragments with different affinities for p53 into p53 tetramer droplets. p53 binds to a specific double-stranded DNA sequence (dsDNA_sp_) using the core domains and to nonspecific dsDNA sequences (dsDNA_nsp_) using the C-terminal domains^26,34,35^. In addition, p53 interacts with single-stranded DNA (ssDNA) using C-terminal domains^36,37^. Titration experiments of p53 binding to three DNA fragments in 50 mM KCl gave dissociation constants of 1.6 nM for dsDNA_sp_^35^, 5.3 nM for dsDNA_nsp_^35^, and 22 ± 1 nM for ssDNA (Supplementary Fig. S2A). The three ATTO488-labeled DNA fragments were recruited into p53 tetramer droplets to different degrees (Supplementary Fig. S2B, C). The average EI values of the DNA fragments were inversely correlated with the dissociation constants, confirming the commitment of the host–guest interactions to recruitment into the droplets (Fig. 3D).

### p53 diffuses slowly in the droplets, irrespective of its oligomeric state and disordered domain removal

**Figure 4:**
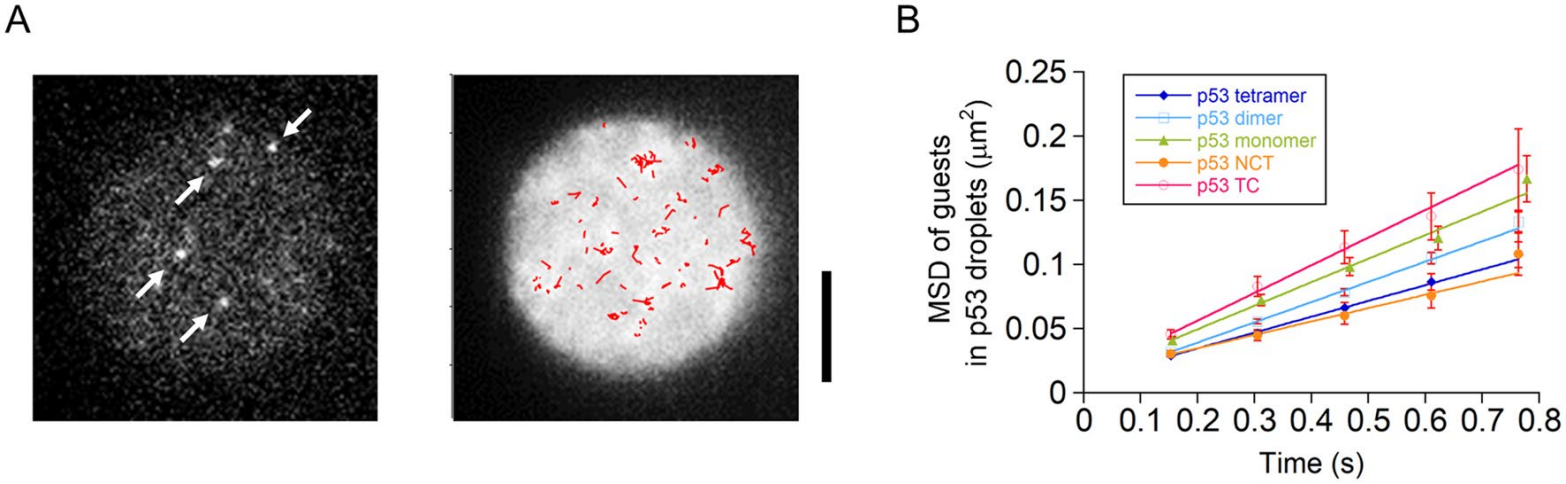
p53 diffuses slowly in the droplets, irrespective of its oligomeric state and droplet-forming disordered domain removal. (**A**) Snapshot (left) and time-averaged (right) fluorescent images of Alexa488-labeled p53 tetramer in a non-labeled p53 tetramer droplet solution. In the left panel, the arrows represent the single p53 molecules. In the right panel, the typical trajectories of single molecules (red) are overlaid. Scale bar denotes 5 μm. (**B**) Mean square displacement (MSD) plots of Alexa488-labeled p53 mutants in non-labeled p53 tetramer droplets. p53 NCT and TC denote mutants lacking the C-terminal domain and N-terminal plus core domains, respectively, maintaining a tetramer. The straight lines show the best-fit linear functions for the MSD data. Error bars denote standard errors.

To elucidate the dynamic property of the labeled p53 in non-labeled p53 tetramer droplets, we conducted single-molecule tracking measurements using HILO illumination. To detect single molecules in the droplet, the concentrations of the labeled guests were reduced to 0.1–0.5 nM in 25 μM non-labeled p53 tetramer solution. We first measured the dynamics of the Alexa488-labeled p53 tetramer. The droplets were observed as slight bright circles above the intensity of the solution due to the fluorescence from many defocused molecules weakly excited by the edge of HILO illumination in the droplets (Fig. 4A). In the droplets, bright moving spots were observed at the single-molecule level at 150 ms time intervals (Fig. 4A and Supplementary movie S1). In contrast, the spots were hardly detected outside the droplets, due to smearing by rapid diffusion in the dilute solution. We tracked the center of the bright spots and obtained 268 traces. The trajectories of individual molecules were plotted in the droplet visualized by time-averaging the images (red traces in the right panel of Fig. 4A). We analyzed the dynamic properties using mean square displacement (MSD) plots. The MSD plots demonstrated a linear relationship with time interval, indicating that the p53 tetramer diffuses inside the droplets (Fig. 4B). The average diffusion coefficient (*D*) was 0.031 ± 0.002 μm^2^/s, identified by fitting the MSD plots with a linear equation with a 4*D* slope.

To investigate the effect of the oligomeric state of p53 on the dynamics, we next measured the Alexa488-labeled p53 dimer (L344A) and monomer (L344P) mutants in the non-labeled p53 tetramer droplets. The linear MSD plots of these mutants confirmed the diffusion within the droplets (Fig. 4B). The average *D* values were 0.039 ± 0.002 and 0.046 ± 0.004 μm^2^/s for the dimer and monomer mutants, respectively, which were slightly higher than that of the p53 tetramer. Although p53 monomerization decreased the EI to 12.5-fold, it retained only a 1.5-fold increase in the average *D*. We next investigated the effect of removing either of the droplet-forming disordered domains on the dynamics. The linear MSD plots of the NCT and TC mutants, which lack C- and N-terminal domains, respectively, confirmed the diffusion in the droplets (Fig. 4B). The average *D* values were 0.026 ± 0.003 and 0.054 ± 0.006 μm^2^/s for the NCT and CT mutants, respectively, which were comparable to or slightly larger than that of the p53 tetramer. Overall, guest p53 diffusion was less sensitive to the reduction of the oligomeric state or droplet-forming disordered domains.

### The diffusion dynamics of guest proteins in p53 droplets depend on the structural properties of guest molecules

**Figure 5:**
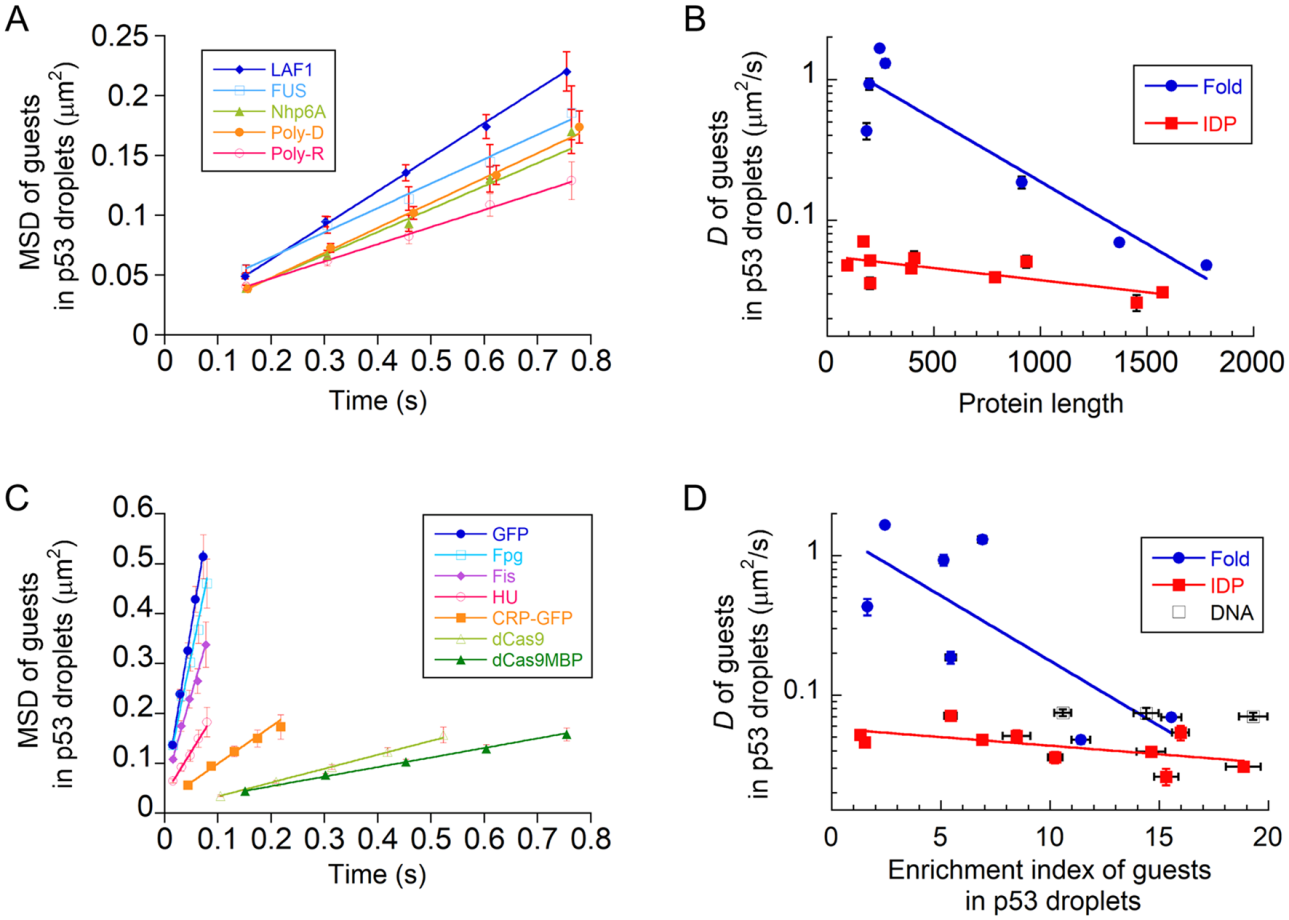
Diffusion of guests in p53 tetramer droplets depend on the structural type and is affected by molecular interaction between host and guests. (**A**) Mean square displacement (MSD) plots of the labeled intrinsically disordered proteins (IDPs) in non-labeled p53 tetramer droplets. (**B**) Protein length dependence of the average *D* values of guest proteins in non-labeled p53 tetramer droplets. (**C**) MSD plots of the labeled folded proteins and GFP in non-labeled p53 tetramer droplets. (**D**) Enrichment index (EI) dependence of the average *D* values of guest proteins in non-labeled p53 tetramer droplets. In panels A and C, straight lines show the best-fitted linear functions for the MSD data. In all panels, error bars denote standard errors. In panels B and D, best-fitted lines for the folded proteins (red) and IDPs (blue) are displayed as a guide for eye.

To elucidate the general dynamic properties of the labeled guest proteins in non-labeled p53 tetramer droplets, we measured several IDPs: LAF1 RGG domain–Alexa488, FUS–MBP–Alexa488, Nhp6A–Atto488, poly-R–Alexa488, and poly-D–Alexa488. In all cases, the MSD plots were linear with respect to the time interval, confirming diffusion (Fig. 5A). The average *D* values ranged from 0.036 ± 0.003 to 0.071 ± 0.004 μm^2^/s, which was within a 2.3-fold increase of that of the p53 tetramer (Table 1). We found that the diffusion of guest IDPs, including p53 mutants, was similar to or slightly higher than that of the host p53, but not significantly dependent on guest protein length (Fig. 5B).

To clarify the effect of different structural properties of guests, we investigated the dynamics of several folded proteins in the p53 tetramer droplets. Contrary to the IDPs, HU–Atto488, Fis–Atto488, CRP–GFP, Fpg–Atto488, and GFP showed linear MSD plots with high slopes (Fig. 5C). The average *D* values were more than sixfold larger than that of the p53 tetramer and widely distributed from 0.18 ± 0.02 to 1.67 ± 0.08 μm^2^/s (Table 1). In contrast, dCas9–Alexa488 and dCas9–MBP–Atto488 diffused slowly, comparable to the IDPs (Fig. 5C and Table 1). The average *D* plots against the protein length demonstrated that the folded proteins diffused faster than the IDPs within 300 residues (Fig. 5B). Next, the heterogeneity of diffusion was examined using the distribution of *D* values of each molecule (Supplementary Fig. S3A). HU–Atto488, Fis–Atto488, Fpg–Atto488, and GFP possessed high and low mobile components, corresponding to more than and less than 0.9 μm^2^/s. In contrast, other folded proteins and IDPs were distributed within a low mobile range (Supplementary Figs. S3A, B). Thus, the folded proteins exhibit widely ranging, complex diffusion dynamics in p53 droplets.

To further characterize the dynamic properties, we plotted the data as functions of the EI values, corresponding to the molecular interaction, and the *D* values in the droplets (Fig. 5D). At EI values < 8, the average *D* values of folded proteins were widely distributed upon the appearance of a high mobile component, whereas IDPs showed similar or slightly fast diffusion to host p53. In contrast, the average *D* values, irrespective of guest structure types, were restricted within 2.4-fold at EI > 8, suggesting that strong host–guest interactions slow diffusion. This is supported by the slow diffusion of three labeled DNA fragments with relatively high affinity in the p53 tetramer droplets (approximately 2.3–2.4-fold larger than that of host p53 tetramer; Fig. 5D and Supplementary Figs. S2D,E).

### MD simulations provide molecular insights into the heterogeneous diffusion of guest proteins, depending on their structural properties

To dissect the linkage between the molecular properties of guest proteins and their diffusion mechanism within liquid droplets, the diffusion of guest proteins within the host p53 condensate was investigated using coarse-grained MD simulations. Six guest proteins were selected for computational study, four of which were folded proteins (Fis, GFP, HU, and Cas9) and the other two were IDPs (poly-R and p53). Prior to simulating the diffusion of these guest proteins, the host p53 tetramer condensate was studied to determine the simulation temperature for forming an equilibrated dense state corresponding to the condensate. The p53 condensate was modeled by 20 tetrameric p53, whose disordered regions are represented as flexible polymers with a single bead per residue, and the folded core and tetramerization (Tet) domains are represented as large spheres (see Methods). Simplifying the representation of the folded p53 domains, which reduces the computational cost of simulating their condensate state, is rationalized by the contribution of the disordered p53 domains in droplet formation^6^.

**Figure 6:**
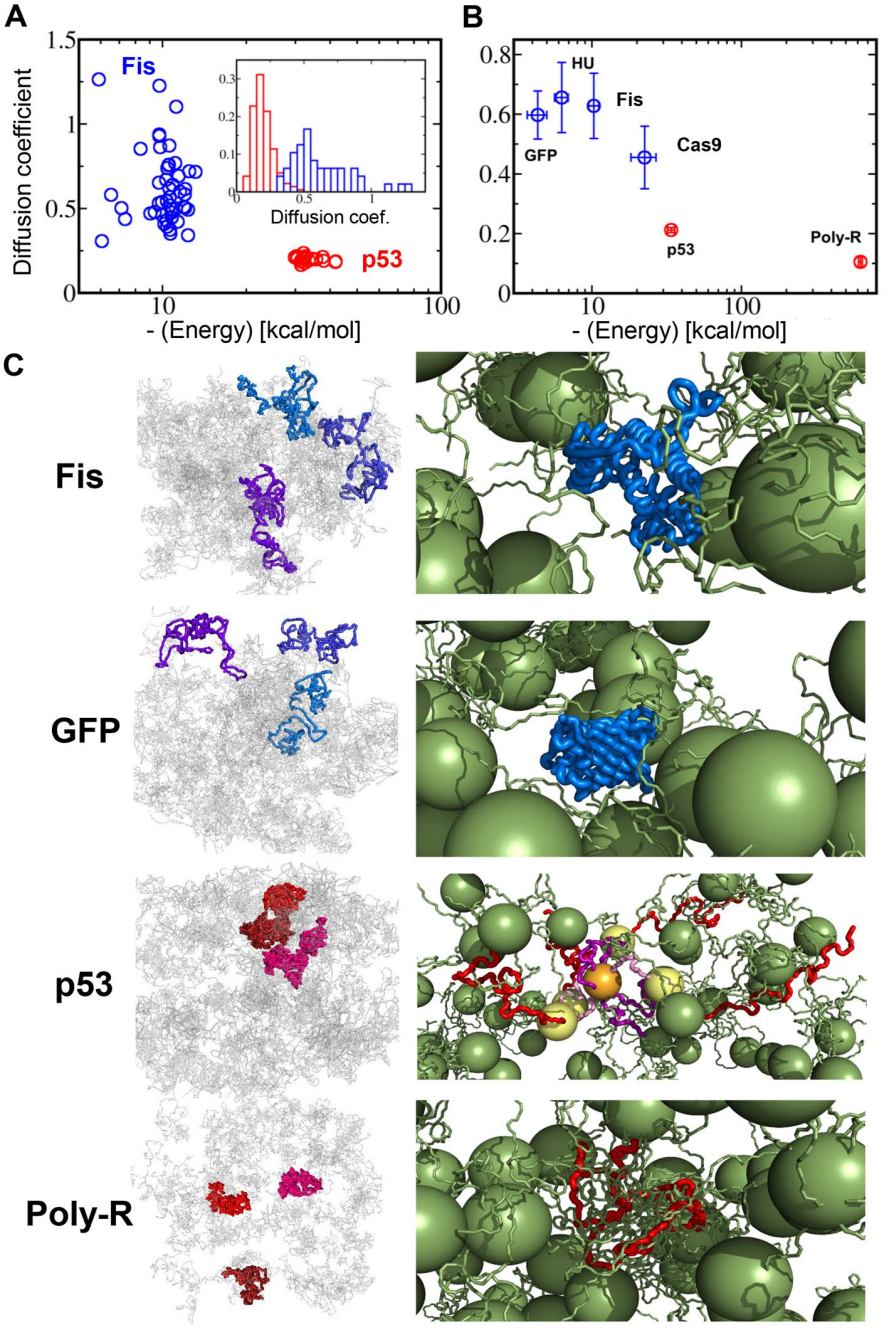
Molecular dynamic (MD) simulations demonstrate different diffusion properties for folded proteins and intrinsically disordered proteins (IDPs) within p53 tetramer condensate. (**A**) Diffusion coefficient plots of guest proteins (Fis and p53) within p53 condensate as a function of the intermolecular energy of these guest proteins with the neighboring p53 tetramers. The inset shows the distribution of the calculated *D* values for Fis and p53. (**B**) Similar plot as in panel (**A**) for four folded proteins (blue) and two IDPs (red). The errors denote the standard deviation reflecting the heterogeneity in the energy and *D* value. (**C**) Projections of three independent trajectories (in reddish or bluish colors) of diffusion for two folded guest proteins (Fis and GFP) and two IDPs (p53 and poly-R) within p53 condensate, illustrating greater restricted diffusion of the IDPs compared with the folded proteins (left panels). Gray lines represent the location of the center of mass of the 20 tetrameric p53 that comprise its liquid-like condensate during the simulations. Snapshot of the guest proteins in p53 condensate illustrate the extensive interactions of the IDPs with p53 via intermolecular interactions between their disordered regions (right panels). The folded proteins can be trapped in voids and interact less tightly with the p53 condensate. The host p53 tetramer are shown in green, with the core and Tet domains as large spheres. The guest folded proteins and IDP are shown in blue and red, respectively. For the guest p53, the core and Tet domains, respectively, are shown in yellow and orange, and the N- and C-terminal disordered domains and linker are shown in red, purple, and pink, respectively.

The diffusion of each guest protein within the p53 condensate was investigated using long MD simulations (Supplementary movies S2 and S3). Figure 6A,B show that the studied folded proteins diffuse on average about two–sevenfold faster than the IDPs, consistent with the experimental results (Fig. 5). Figure 6C shows three representative trajectories of translational movement of selected guest proteins within the p53 condensate, illustrating the extensive diffusion of Fis and GFP in comparison to p53 and poly-R. To understand the origin of the different diffusion coefficients of the studied proteins, the calculated *D* values were plotted against the intermolecular energy between the guest proteins and the surrounding p53 tetramers. This indicates that slow diffusion is linked to stronger intermolecular energy in the guest IDPs than in the guest-folded proteins (Fig. 6A,B). The qualitative agreement between the results presented in Fig. 6B, 5D supports that the experimentally measured EI is governed by intermolecular interactions between the guest proteins and the surrounding host proteins within the condensate.

In addition to the increased average *D* values of the folded proteins, their diffusion also becomes heterogeneous, as reflected by the broader distribution of the *D* values for Fis than that for p53 (Fig. 6A) and the large standard deviation of the distributions of *D* of all the folded proteins (Fig. 6B). This observation is qualitatively consistent with the experimental data (Supplementary Fig. S3). The increased heterogeneous diffusion of Fis, GFP, HU, and Cas9 is attributed to different interactions with the surrounding environment provided by neighboring p53 molecules in the condensate. These folded proteins diffuse while locating in voids of various sizes within the condensate (as well as diffusion on the droplet surface), while interacting weakly and non-homogenously with the disordered p53 domains (right panels of Fig. 6C and Supplementary movie S2). In contrast, the guest IDPs, p53 and poly-R adjust their structures to interact tightly with the disordered p53 domains, regardless of their location in the p53 condensate, which results in slow homogenous diffusion (right panels of Fig. 6C and Supplementary movie S3).

## Discussion

Fluorescence measurements of the guest protein series indicated that the recruitment properties of folded and disordered guest proteins were similar, which is different from the high tendency of IDPs to form droplets as host proteins^7,38^. Structure-indistinguishable recruitment may be caused by structural host p53 adaptation to associate with the guest proteins, albeit folded host proteins, via a common intermolecular interaction. One of the key interactions is the cation–π interaction between R and Y residues. This is supported by the correlation between the number of R and Y residues and the recruitment tendency of various FUS family proteins in FUS droplets^10^. Moreover, poly-R is recruited into FUS droplets and promotes these formations^33^. Similarly, our results showed a moderate correlation between recruitment tendency and the number of R and Y residues, highlighting the importance of cation–π interactions in recruitment (Fig. 3C). The other key interaction is electrostatic interactions. The recruitment tendency was moderately correlated with the total number of charges (Fig. 3B). The host p53 tetramer possesses four oppositely charged disordered domain sets that interact with guest proteins via electrostatic interactions. As the protein length increases, the number of cation–π and electrostatic interactions between the host and guest proteins increase, resulting in increased recruitment. This is highlighted by the lack of significant correlation between the EI values and the relative content of R plus Y residues or charges. The protein length dependence of the recruitment does not concur with the absence of molecular weight dependence observed in Ddx4 droplets^14^, which may reflect differences in solvent accessibility of residues in folded structures. The coupling between the intermolecular interaction and recruitment tendency is supported by a theoretical study^39^, DNA recruitment in this study, and artificial host–guest systems^13^ (Fig. 3D). Thus, we propose that the cation–π and electrostatic interactions participate in guest protein recruitment in liquid droplets, providing clues for predicting guest protein recruitment into membraneless organelles, even if specific host–guest interactions are missing.

The dynamics of guest proteins in liquid droplets are more complicated than in a uniformly diluted solution, which is determined by the viscosity and molecular size following the Stokes–Einstein equation. We found that guest IDPs exhibited homogeneous, slow dynamics in the liquid droplets (Fig. 5B,D). The guest IDPs interact with host molecules within the region covered by the extended disordered regions, which is larger than that of globular folded proteins. In addition, the IDPs adapt these structures to tightly interact with neighboring host molecules (Fig. 6C). Disordered host protein regions also assist host–guest interactions through their flexible structures. One may consider that the weakening of intermolecular interactions, corresponding to a low EI, is expected to facilitate guest IDP movement in liquid droplets. However, after dissociation from the host molecule, the extended and adaptable IDP structure likely enables the immediate formation of a new interaction with the neighboring host molecules, resulting in non-significant or slightly enhanced movement (Fig. 5D). In other scenarios, the guest IDPs interact with the first host molecule and then interact with the second host molecule before releasing the first molecule, and then release the first one and interact with the third host molecule, similar to moving on monkey bars. Accordingly, we propose that the extended and adaptable structure of guest IDPs causes these slow dynamics in liquid droplets, which is different from the dynamics of folded guest proteins.

Unlike the IDPs, the folded guest proteins exhibited a wide range of diffusion depending on their size and intermolecular interactions (Fig. 5B,D). This is consistent with the observation that the modulation of intermolecular interactions affect the dynamic behavior of other LLPS systems^10,40,41^. Furthermore, heterogeneous dynamics were observed in RNA–protein LLPS systems^23,24^, supporting the complex dynamics of the droplets. The MD simulations suggest that folded proteins diffuse within the voids of the liquid droplet while interacting weakly with neighboring host proteins, causing heterogeneous dynamics (Fig. 6C). The presence of voids inside the droplets has also been reported in other studies^15–17^. In contrast to IDPs, folded proteins cannot adapt their structures to form tight interactions with host molecules. The different structural properties of the IDPs and folded proteins are attributed to the different dynamic behaviors of the droplets.

## Materials and methods

### p53 mutants

We prepared the p53 tetramer as well as the NCT, TC, and dimer mutants as described previously^6,25,32,35^. For the p53 tetramer, a thermostable and cysteine-modified human p53 mutant (C124A, C135V, C141V, W146Y, C182S, V203A, R209P, C229Y, H233Y, Y234F, N235K, Y236F, T253V, N268D, C275A, C277A, K292C) was used^25^. The TC mutant corresponds to residues 293–393 of the tetramer, with an additional N-terminal cysteine^25^. The NCT mutant corresponds to residues 1–363^31^. The dimer mutant corresponds to L344A of the tetramer sequence^6^. The monomer mutant (L344P) was prepared using the tetramer. The monomer mutant gene in pGEX-6P-1 was generated using a PrimeSTAR Mutagenesis Basal Kit (TaKaRa). These p53 mutants were expressed and purified as previously described^25,31^. We confirmed the oligomeric state of the p53 monomer L344P mutant, dimer L334A mutant, and tetramer using a gel filtration column (Superdex 200; GE Healthcare).

### Other guest samples

The RGG domain of *Caenorhabditis elegans* LAF-1 (residues 1–168) with an engineered Cys and 6 × His tag at the C-terminus was expressed and purified as previously described^42^, but with some modifications. The RGG domain gene was artificially generated (Eurofins), and Cys was inserted using a KOD-Plus Mutagenesis Kit (TOYOBO). For *human* FUS fused to MBP, 6 × His–MBP–TEV–FUS with a C-terminal engineered Cys was expressed and purified without 6 × His–MBP tag cleavage, as previously described^33^. *Escherichia coli* Fis Q21C mutant and *Saccharomyces cerevisiae* Nhp6A 2-Cys mutant (containing Cys at residue 2 and the C-terminal end) were expressed and purified without tags, as previously described^43,44^. For dCas9 (deactivated Cas9 from *Streptococcus pyogenes*) samples, 10 × His–MBP–TEV–dCas9 (M1C, D10A, C80S, H840A, C574S; Addgene 60815) was expressed and purified with or without 10 × His–MBP tag cleavage as described by Trishit et al.^45^. For GFP, the opt-mutant (S30R, Y39I, F64L, S65T, F99S, N105K, E111V, I128T, Y145F, M153T, V163A, K166T, I167V, I171V, S205T, A206V) with a C-terminal 6 × His tag was expressed and purified with a tag as previously described^46^. For Venus, the opt-mutant (S30R, Y39I, F46L, F64L, S65G, V68L, S72A, F99S, N105K, E111V, I128T, Y145F, M153T, V163A, K166T, I167V, I171V, S175G, T303Y, S205T, A206V) with a C-terminal 6 × His tag was expressed and purified with a tag as previously described^46^. *Lactococcus lactis* Fpg and HU containing an engineered Cys at the C-terminus were overproduced and purified as previously described^47,48^. For *E. coli* CRP fused to GFP, purified CRP with N-terminal eGFP was provided by Sridhar Mandali and Reid C. Johnson (UCLA).

For DNA samples, we used the following DNA fragments with a 5’-terminal Atto488. The specific DNA sequence was 5’-ATCAGGAACATGTCCCAACATGTTGAGCTC-3’, which corresponds to the p21 5’ promoter site^35^. The nonspecific DNA sequence was 5’-AATATGGTTTGAATAAAGAGTAAAGATTTG-3′^35^. The ssDNA sequence was 5’-ATCAGGAACATGTCCCAACATGTTGAGCTC-3′^6^. These DNA fragments were purchased from Sigma-Aldrich.

### Labeling with fluorophores

Except for the RGG domain of LAF-1, CRP–GFP, GFP, and Venus were labeled with Atto488 (ATTO-TEC) or Alexa488 (Thermo Fisher) using maleimido chemistry and were then purified with a cation exchange, heparin, or gel filtration column. The N-terminus of poly-R (poly-L-R with 15–70 kDa and median 200-mer; Sigma-Aldrich) and poly-D (poly-L-D with 23 kDa and 200-mer; ALAMANDA polymers) were labeled with Alexa488 using succinimidyl ester chemistry and purified using gel filtration^33^. The N-terminus of the RGG domain of LAF-1 was labeled with Alexa488 using SDP chemistry and purified using HPLC.

### Recruitment measurements

For recruitment measurements, solutions containing 25 μM non-labeled p53 tetramer, 100 mM Tris, 150 mM NaCl, 1 mM dithiothreitol (DTT), 100 mg/mL dextran (MW 45,000–65,000; Sigma-Aldrich), and 100 nM fluorescent samples at pH 7.4 were used. Droplet formation was triggered by tenfold dilution of a non-labeled p53 tetramer stock solution containing 450 mM NaCl at pH 7.5. The solutions were incubated at 20 °C for at least 5 min. The sample solutions were cast on a coverslip and covered with a glass slide (Matsunami Glass) through a 100-μm-thick double-sided tape. The coverslip was cleaned with a solution containing H_2_O_2_, 30% NH_3_, and H_2_O in a 1:1:1 ratio before use. We used an inverted fluorescence microscope (IX-73; Olympus) with a total internal reflection fluorescence unit (IX3RFAEVAW; Olympus)^43,44^. An objective lens (NA = 1.49) was illuminated using a 488-nm laser with a highly inclined thin illumination geometry. Fluorescence collected by the objective lens was detected using an EM-CCD camera (iXon Ultra 888; Andor). To prevent photo-bleaching of the fluorescent samples, we used 0.15 mW laser power. Images were acquired at 20 °C. Using ImageJ software, we calculated the average fluorescence intensities of individual droplets (*I*_droplet_) and of solutions (*I*_solution_) near the droplets with background substitution and obtained EI values by dividing *I*_droplet_ by *I*_solution_.

### Single-molecule measurements

We used solutions containing 25 μM non-labeled p53 tetramer, 100 mM Tris, 150 mM NaCl, 1 mM DTT, 100 mg/mL dextran, and 0.1–0.5 nM fluorescent samples at pH 7.4. To prevent fluorescent sample adsorption, we coated the coverslip with 0.5% 2-methacryloyloxyethyl phosphorylcholine (MPC) polymer (Lipidureμ-CM5206; NOF Corp.) in ethanol^49^. The above-mentioned microscope was used with a laser power of 3–5 mW. Time courses of images were recorded at 15–150 ms time intervals after reducing the number of observable molecules in the droplets by photo-bleaching for 1–2 min. The fluorescent spots of single molecules were tracked from sequential images using ImageJ software with the plugin ‘Particle track and analysis’. We selected trajectories with at least six consecutive points, and MSDs were calculated from all pairs of two-dimensional positions of a molecule at each time interval for all trajectories using our in-house program, with some modifications^25,44^. Average *D* values were calculated by fitting the slopes of the MSD plots (five data points) using 4*D*. We calculated *D* values for each molecule using MSDs of the initial five displacement steps of a single molecule divided by a four-fold time interval^50,51^.

### MD simulations

A simple tetrameric p53 model was used to study its liquid-like condensate, in which a p53 monomer contains two folded domains (core and Tet) and three disordered regions. Because the disordered p53 domains play an important role in p53 LLPS^6^, the core and Tet domains were modeled as spheres represented by a single bead with 21 and 15 Å radii, respectively, representing the dimensions of their crystal structures. The disordered p53 regions were modeled at the amino acid resolution, where each residue was modeled using a single bead. When modeling the p53 tetramer, the bead that represents the Tet domain was linked to four linkers and four C-terminal disordered domains. The four linkers were each connected to core and N-terminal disordered domains. The condensate was studied by simulating 20 copies of tetrameric p53 in a cubic box with a length of 120 nm. The diffusion of guest proteins in the p53 condensate was studied computationally for four folded proteins (GFP [PDB 5B61], Fis [PDB 3IV5], HU [PDB 5LVT], and Cas9 [PDB 4CMP]), and two IDPs (p53 and poly-R with 200 residues). The diffusion of p53 was tracked by following each of the p53 tetramers comprising the condensate. All simulations were initiated when the guest proteins were close to the surface of the p53 condensate.

In our model, the liquid-like p53 condensate, as well as the interactions between p53 and the guest proteins, were governed by electrostatic and short-range hydrophobic interactions between the charged (K, R, D, and E) and hydrophobic (V, F, L, N, Q, I, and W) residues, respectively. These interactions were applied both intra- and inter-molecularly, using a recent model that quantifies the role of short- and long-range interactions in LLPS^52,53^. The electrostatic interactions were modeled using the Debye-Hückel formalism, and short-range hydrophobic interactions were modeled by Lennard–Jones interactions. In addition, the intramolecular interactions that maiantin the structure of the folded guest proteins were introduced by Lennard–Jones interactions with a strength of *ε* = 1.5 kcal/mol in order to preserve their folded state. The strength of the short-range interaction between the disordered regions was selected to represent a realistic IDP behavior. A value of *ε* = 0.2 kcal/mol showed the best correlation between the calculated and measured radius of gyration of several IDPs^53^.

Starting from an initial configuration of the guest proteins in the dense phase of p53, eight independent trajectories were simulated for 8 × 10^6^ steps for each guest protein using the Langevin equation. The simulations were performed at 0.02 M salt concentration and at *T* = 0.4, which was lower than the critical temperature of p53 LLPS. Translational diffusion coefficients were measured as the slope of the MSD of the center of mass of the studied proteins. The periods during which the proteins were dissociated from the condensate were excluded when calculating the *D* values of the guest proteins within the p53 condensate. To understand the heterogeneous dynamics within the condensate, each trajectory was divided into eight fragments with 10^6^ timesteps, and the *D* value was calculated for each of these fragments. Additional methodological details are provided in the Supplementary Material.

## Supplementary Information

Supplementary Information 1.

Supplementary Video 1.

Supplementary Video 2.

Supplementary Video 3.
