# Supplementary material for: Molecular principles of recruitment and dynamics of guest proteins in liquid droplets
Source: Sci Rep. 2021 Sep 29;11:19323. doi: 10.1038/s41598-021-98955-0 (PMC8481498; doi:10.1038/s41598-021-98955-0)
Supplement: Supplementary file 1 — Supplementary Information 1. [file 41598_2021_98955_MOESM1_ESM.docx]

Supplementary Information for

Molecular principles of recruitment and dynamics of guest proteins in liquid droplets

*Corresponding author: Kiyoto Kamagata

Institute of Multidisciplinary Research for Advanced Materials, Tohoku University, Katahira 2-1-1, Aoba-ku, Sendai 980-8577, Japan

TEL: +81-22-217-5843/FAX: +81-22-217-5842

e-mail: kiyoto.kamagata.e8@tohoku.ac.jp


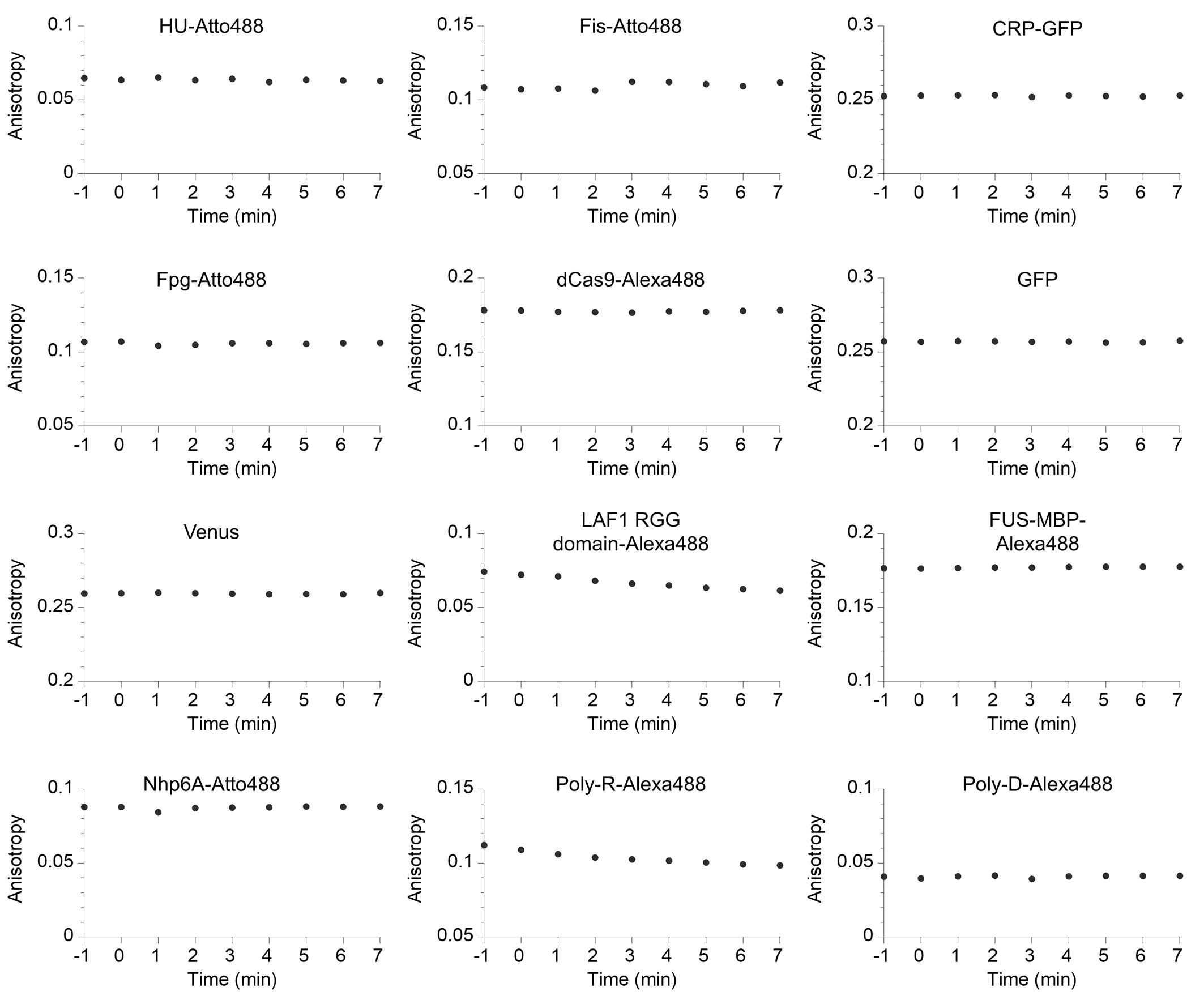


**Fig. S1.** Time course of fluorescence anisotropy of labeled guest proteins following addition of non-labeled p53 for testing interactions between host and guest proteins under no droplet condition. The measurements were conducted in a solution containing 5 nM labeled protein, fluorescent protein, or polymer, 100 mM Tris, 150 mM NaCl, 1 mM DTT, and 0.2 mg/mL BSA at pH 7.5 and 25 °C. The 100 nM of non-labeled p53 was added at the beginning of the experiment (0 min).


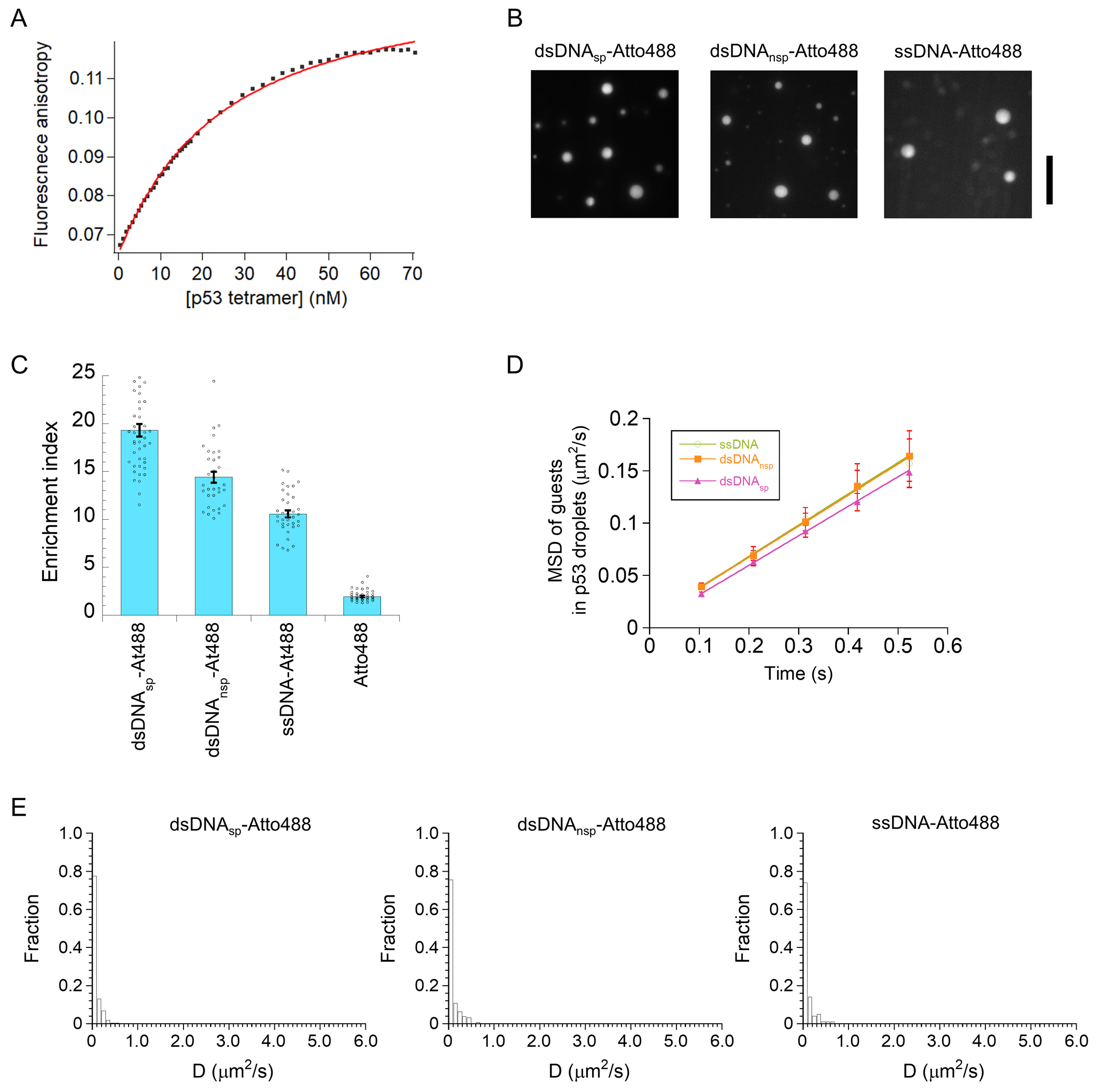


**Fig. S2.** (A) Fluorescence anisotropy changes for the association between p53 tetramer and single-stranded DNA sequence conjugated with 6-FAM. The titration was conducted in a solution containing 20 mM HEPES, 50 mM KCl, 2 mM MgCl_2_, 0.5 mM EDTA, 1 mM DTT, 0.2 mg/mL BSA, and 5 nM ssDNA at pH 7.9 and 25 °C. The solid curve represents the best-fitted curve based on the equation assuming one-to-one binding (1). (B) Fluorescent images of Atto488-labeled DNA fragments and Atto488 in the non-labeled p53 tetramer droplet solution. Scale bar denotes 20 μm. (C) Enrichment indices of the labeled DNA fragments in the non-labeled p53 tetramer droplet. The errors denote the standard errors. Significant differences in average EI values between three DNA fragments and Atto488 were confirmed using Welch’s t test with α = 0.05. (D) MSD plots of Atto488-labeled DNA fragments in the droplets of non-labeled p53 tetramer. Straight lines show the best fitted linear functions for the MSD data. Error bars denote standard errors. (E) Distribution of diffusion coefficients of individual molecules of Atto488-labeled DNA fragments in the droplets of non-labeled p53 tetramer.


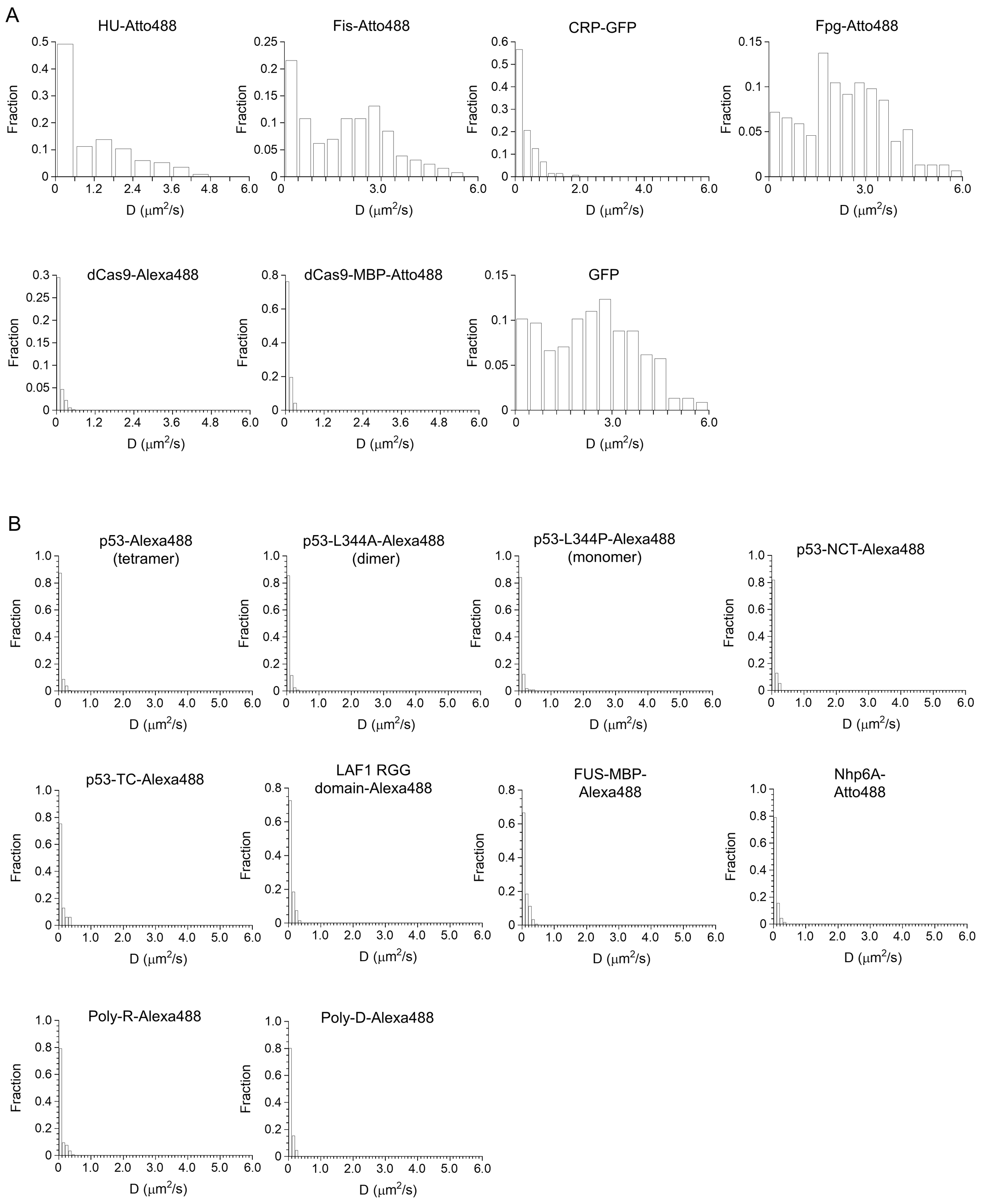


**Fig. S3.** Distribution of diffusion coefficients of individual molecules of structured proteins (A) and IDPs (B) in droplets of non-labeled p53 tetramer.

**Supplementary movie S1.** Movie showing alexa488-labeled p53 (tetramer) in droplets of non-labeled p53 tetramer using single-molecule fluorescence microscopy. Single white dots represent labeled p53 molecules.

**Supplementary movie S2.** Video showing the folded guest protein Fis moving in a p53 condensate using coarse-grained molecular dynamics simulation. The host p53 tetramer is shown in green, with the core and Tet domains as large spheres. The Fis molecule is shown in blue.

**Supplementary movie S3.** Video showing a guest p53 moving in a p53 condensate using coarse-grained molecular dynamics simulation. The host p53 tetramer is shown in green, with the core and Tet domains as large spheres. For the guest p53, the core and Tet domains are shown in yellow and orange, respectively, and the disordered domains and linker are shown in red.

**Supplementary text**

**Detailed methodology for molecular dynamics simulations.** We first constructed a simple tetrameric p53 model to study its liquid-like condensate. Each p53 monomer contains two folded domains (core and Tet) and three disordered regions. The tetramer was formed via the Tet domain. Since it was shown experimentally that the disordered domains of p53 play a more important role in LLPS of p53 (2), we modeled the folded domains and disordered regions of p53 with different degrees of coarse-graining. The core and Tet domains were modeled as spheres represented by a single bead with radii of 21 Å and 15 Å, respectively, representing the dimensions of the crystal structures. The disordered regions of p53 were modeled at the amino acid resolution, where each residue was modeled by a single bead. Modeling the core and Tet domains as a single bead was beneficial for reducing the computational cost of the simulations for very large systems, including multiple copies of p53. To model the p53 tetramer, the bead representing the Tet domain was linked to four linkers and four C-terminal disordered domains. The four linkers were each connected to a core and N-terminal disordered domain. The condensate was studied by simulating 20 copies of tetrameric p53 in a cubic box with a length of 120 nm. The properties of the p53 condensate were studied at several temperatures to quantify its phase diagram. To enhance the equilibration of the p53 condensate, the simulations were initiated from a configuration where the p53 tetramers were relatively packed.

The diffusion of guest proteins in the p53 condensate was studied computationally for four folded proteins [GFP (PDB 5B61), Fis (PDB 3IV5), HU (PDB 5LVT), and Cas9 (PDB 4CMP)] and two IDPs (p53 and poly-R with 200 residues). The diffusion of p53 was tracked by following each p53 tetramer comprising the condensate. All these simulations were initiated when the guest proteins were close to the surface of the p53 condensate. In the simulations, the guest proteins often exited the condensate and then re-entered after some time. When calculating the *D* values of the guest proteins within the p53 condensate, the periods when the proteins were dissociated from the condensate were excluded.

In our model, the liquid-like condensate of p53 was governed by electrostatic interactions and short-range hydrophobic interactions. The electrostatic interactions were between the charged residues (K, R, D, and E), while the hydrophobic interactions were between hydrophobic residues (V, F, L, N, Q, I, and W). These interactions were applied to both intra- and inter-molecular interactions following a model that quantified the role of short- and long-range interactions in LLPS (3, 4). The electrostatic interactions were modeled using the Debye-Hückel formalism as follows: $E_{electrostatic}=K_{Coulomb}B(\kappa)\sum_{i,j} \frac{q_{i}q_{j}e^{-\kappa r_{ij}}}{\varepsilon r_{ij}}$, where $q_{i}$ and $q_{j}$ denote the charges of the *i*^th^ and j^th^ beads, respectively, $r_{ij}$ denotes the inter-bead distance, $\varepsilon$ is the dielectric constant of the solvent, and $K_{Coulomb}=4\pi\varepsilon_{0}=$332 kcal/mol. The term $B(\kappa)$ is a function of the salt concentration and the radius ($a$) of ions generated due to the dissociation of the salt, and is given by $B\left( \kappa\right)=\frac{e^{\kappa a}}{1+e^{\kappa a}}$. According to the Debye-Hückel theory, the range of electrostatic interactions of an ion is of the order $\kappa^{-1}$, which is called the Debye screening length. The Debye screening length is associated with the ionic strength as follows: $\kappa^{2}=\frac{8\pi N_{A}e^{2}\rho_{A}I}{1000\varepsilon k_{B}T}$, where $N_{A}$ is the Avogadro number, *e* is the charge of an electron, $\rho_{A}$ is the solvent density, *I* is the ionic strength of the medium, $k_{B}$ is the Boltzmann constant, and T is the temperature.

The short-range hydrophobic interactions were modeled using Lennard-Jones interactions as follows: $E_{Short-range}=4\varepsilon\left[ \left( \frac{\sigma_{ij}}{r_{ij}} \right)^{12}-\left( \frac{\sigma_{ij}}{r_{ij}} \right)^{10} \right]$ , where $\sigma_{ij}$ denotes the optimal distance between beads *i* and *j* that are in contact with each other, and $\sigma_{ij}$ was chosen as $7Å$. $\varepsilon$ is the strength of the short-range interaction, and its value was selected to represent realistic IDP behavior. As reported in our previous study, a value of *ε*=0.2 kcal/mol showed the best correlation between the calculated and measured radius of gyration of several IDPs (4).

The interactions between the guest proteins and the p53 molecules forming the condensates were also modeled using electrostatic and hydrophobic interactions, based on parameters identical to those that define the condensate. In addition, the intramolecular interactions that maintain the structure of the folded guest proteins were introduced using Lennard-Jones interactions with a strength of *ε*=1.5 kcal/mol in order to preserve their folded state.

Starting from an initial configuration of the guest proteins in the dense phase of p53, eight independent trajectories were simulated for 8 × 10^6^ steps for each guest protein using the Langevin equation. The simulations were performed at a salt concentration of 0.02 M and at *T*=0.4, which was lower than the critical temperature of p53 LLPS. Translational diffusion coefficients were measured as the slope of the MSD of the centers of mass of the studied proteins (*x*(*t*), *y*(*t*), *z*(*t*)), which in three dimensions satisfies the equation *D*=$\left\langle\left( x\left( t \right)-x(0) \right)^{2} \right\rangle+\left\langle\left( y\left( t \right)-y(0) \right)^{2} \right\rangle+\left\langle\left( z\left( t \right)-z\left( 0 \right) \right)^{2} \right\rangle/6t$. To understand the heterogeneous dynamics within the condensate, each trajectory was divided into eight fragments with 10^6^ timesteps, and the *D* value was calculated for each of these fragments.

References

1. A. Murata*, et al.*, One-dimensional search dynamics of tumor suppressor p53 regulated by a disordered C-terminal domain. *Biophys. J.* 112, 2301-2314 (2017).

2. K. Kamagata*, et al.*, Liquid-like droplet formation by tumor suppressor p53 induced by multivalent electrostatic interactions between two disordered domains. *Sci. Rep.* 10, 580 (2020).

3. M. K. Hazra, Y. Levy, Charge pattern affects the structure and dynamics of polyampholyte condensates. *Phys. Chem. Chem. Phys.* 22, 19368-19375 (2020).

4. M. K. Hazra, Y. Levy, Biophysics of Phase Separation of Disordered Proteins Is Governed by Balance between Short- And Long-Range Interactions. *J. Phys. Chem. B* 125, 2202-2211 (2021).
